# The Changing Landscape of Intravenous Thrombolysis for Acute Ischaemic Stroke

**DOI:** 10.3390/jcm13195826

**Published:** 2024-09-29

**Authors:** Jack Donaldson, Joel Winders, Yassar Alamri, Dhara Knight, Teddy Y. Wu

**Affiliations:** 1 Department of Neurology, Christchurch Hospital, Christchurch 8011, New Zealand; jack.donaldson@cdhb.health.nz (J.D.); jd.winders@gmail.com (J.W.); dhara.knight@cdhb.health.nz (D.K.); 2 Department of Medicine, University of Otago, Christchurch 8011, New Zealand

**Keywords:** thrombolysis, stroke, tenecteplase, alteplase, outcome

## Abstract

Intravenous thrombolysis remains the most accessible and effective reperfusion therapy available to patients with acute ischaemic stroke. Treatment with intravenous thrombolysis improves the odds of favourable functional outcome with the unacceptably low risk of haemorrhagic complications. Even in the current era of endovascular thrombectomy, intravenous thrombolysis remains the backbone of acute stroke treatment due to its accessibility and relative ease of administration. Since intravenous alteplase was first approved for acute ischaemic stroke in the mid 1990s, there have been significant advances in expanding the indication and time window for treatment, in addition to transitioning towards tenecteplase use for stroke thrombolysis. In this review, we will provide a narrative on the use of thrombolysis in acute ischaemic stroke including an up-to-date discussion on recent advances in thrombolytic therapy.

## 1. Introduction

Intravenous thrombolysis remains the most accessible reperfusion therapy for acute ischaemic stroke despite recent advances in endovascular thrombectomy (EVT). However, as EVT can only be offered to the 30–40% of stroke patients with large vessel occlusion, systemic thrombolysis remains a vital first line treatment.

Intravenous alteplase, a recombinant tissue-type plasminogen activator (r-tPA), has been the standard thrombolytic agent for three decades since it was first proven to be effective in the mid-1990s. It is administered in a split dose fashion with a 10% bolus of total dose (0.9 mg/kg) followed by an hour-long infusion of the remaining dosage. Since the initial landmark NINDS (National Institute of Neurological Disorders and Stroke) trial with a 3 h time window [1], there has been a progressive expansion of the window of eligibility, transitioning from a time-based selection approach to a tissue-based approach using advanced imaging [2].

Tenecteplase, a genetic mutant of alteplase, has been long used for ST segment myocardial infarction [3]. Tenecteplase has a longer half-life than alteplase and is pragmatically advantageous due to single bolus administration. Tenecteplase at 0.25 mg/kg has been shown to improve early recanalization compared with alteplase in the setting of large vessel occlusion undergoing EVT, and several large clinical trials have demonstrated non-inferiority to alteplase when used within the standard 4.5 h time window [4,5,6,7,8]. Using advanced imaging selection, tenecteplase can also be administered to select patients with large vessel occlusion up to 24 h from stroke onset [9]. Although European Stroke Organisation and Stroke Foundation of Canada guidelines endorse the use of tenecteplase in acute ischaemic stroke [10,11], its use remains off-label in most parts of the world.

We aim to provide a narrative review of the role of thrombolysis in acute ischaemic stroke and to provide an up-to-date summary of the recently completed clinical trials supporting the changing of the guard to tenecteplase.

## 2. Time Window for Thrombolysis—From Time Clock to Tissue Clock

Intravenous thrombolysis within 4.5 h of symptom onset is considered the routine time window without the need for advanced imaging selection with either computed tomography (CT) perfusion or magnetic resonance imaging (MRI). However, this restrictive time-based criteria excludes a significant proportion of patients due to unwitnessed onset or uncertain onset due to aphasia or waking with symptoms and those presenting beyond 4.5 h. Because of this, it would preclude patients living at distance from a thrombolysis-capable stroke centre due to transport delays.

**Figure 1:**
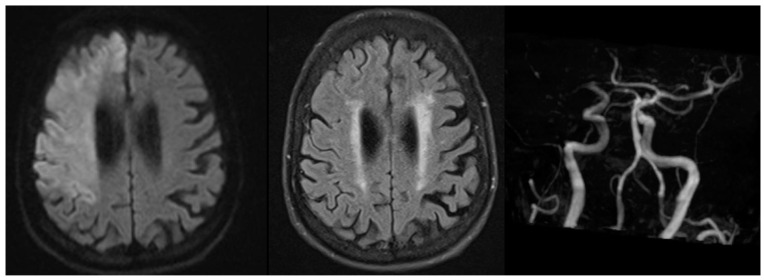
Example of MRI DWI-FLAIR mismatch in a patient with occlusion of right terminal internal carotid artery.

Several randomised clinical trials have assessed the benefit of an imaging-based selection approach to manage these patients with intravenous thrombolysis. The Efficacy and Safety of MRI-Based Thrombolysis in Wake-Up Stroke (WAKE-UP) trial [3] used the concept of DWI-FLAIR mismatch (Figure 1) and randomised patients with wake-up stroke or stroke of unknown onset time to either placebo or intravenous alteplase on the premise that the stroke onset was recent given lack of corresponding FLAIR changes. The trial included 503 patients, with 254 randomised to alteplase and 249 to placebo. Despite not reaching its pre-specified target of 800 due to cessation of funding, alteplase treatment was significantly associated with favourable functional outcomes (mRS of 0–1) at 90 days compared to the placebo group (53.3% vs. 41.8%, adjusted OR1.61; 95% CI 1.09 to 2.36, *p* = 0.02.). Although there was a numerically higher number of symptomatic intracranial haemorrhages in the alteplase group, this was not statistically significant (2.0% vs. 0.4%, aOR 4.95; 95% CI 0.57 to 42.87, *p* = 0.15). Similar trends were seen for rates of death at 90 days (aOR 3.38; 95% CI 0.92–12.52, *p* = 0.07).

However, the major limitation of using an MRI imaging approach is that MRI may not be accessible particularly in middle- and lower-income countries. Ma et al. [4] reported the results of the Extending the Time for Thrombolysis in Emergency Neurological Deficits (EXTEND) trial, which was a placebo-controlled trial randomising patients to treatment within 9 h (9 h in this study refers to within 9 h of the midpoint between falling asleep and waking with stroke symptoms) of last known well or wake-up stroke. Most patients in this trial were screened by CT perfusion imaging and were eligible if they had a perfusion mismatch. The study was terminated early due to the publication of the WAKE-UP trial and enrolled 225 patients out of the planned 310 patients. Patients allocated to intravenous thrombolysis were more likely to achieve functional independence at 90 days (35.4% vs. 29.5%, adjust risk ratio 1.44, 95% CI 1.01–2.06, *p* = 0.04). There was a trend towards increased risk of symptomatic haemorrhage (6.2% vs. 0.9%, *p* = 0.05).

A systematic review and individual patient data meta-analysis by Campbell et al. [5] with data from three clinical trials utilising perfusion imaging selection to treat patient beyond 4.5 h (EXTEND, ECASS4: ExTEND (European Cooperative Acute Stroke Study − 4: Extending the time for thrombolysis in emergency neurological deficits) [6] and EPITHET (Echoplanar Imaging Thrombolytic Evaluation Trial) [7]) included 414 patients and showed that alteplase treatment was associated with a higher rate of excellent functional outcome (mRS 0–1) at 3 months (36% vs. 29%, OR 1.86; 95% CI 1.15 to 2.99, *p* = 0.01). Rates of symptomatic intracranial haemorrhage were comparable (~5%) to the 3–4.5 h time window for alteplase in the ECASS III trial [12].

The major limitation of the extended time window trials was that most patients were recruited prior to the era of thrombectomy, and patients with LVO (~31% in WAKE-UP and 61% in the meta-analysis by Campbell et al. [13]) in the extended time window would likely have been eligible for endovascular thrombectomy with the current approach.

Two further clinical trials assessing the effect of 0.25 mg/kg tenecteplase thrombolysis between 4.5 and 24 h in patients with confirmed anterior circulation LVO have been published.

Both the Thrombolysis in Imaging-eligible, Late-window Patients to Assess the Efficacy and Safety of Tenecteplase (TIMELESS) trial and the Tenecteplase Reperfusion Therapy in Acute Ischemic Cerebrovascular Events–III (TRACE-III) trial randomised patients with proven LVO with perfusion mismatch to either tenecteplase or best medical therapy [9,14]. The major difference between TIMELESS and TRACE III was that EVT patients were included in TIMELESS while TRACE III only enrolled patients without access to EVT.

TIMELESS [14] randomised 458 patients to tenecteplase therapy or placebo, and EVT was performed in 77.3% of the entire cohort. The primary endpoint was ordinal score on the modified Rankin Scale, and there was no difference between the two arms, with a median score of 3 in each arm (*p* = 0.45). There were also no differences in functional outcome as a dichomtized outcome (90-day modified Rankin 0–2, 46% vs. 42.4%) or on the ordinal scale. However, in the subgroup analysis, tenecteplase resulted in improved functional outcomes for patients with M1 occlusion in the primary outcome with adjust common OR on modified Rankin scale score of 1.59 (95% CI 1.00–2.52) and with more achieving 90-day independence 45.9% vs. 31.4% (aOR, 2.03; 95% CI, 1.14 to 3.66). The rate of symptomatic intracranial haemorrhage was 3.2%.

TRACE-III [9] was conducted in China and included only Chinese patients, with the primary outcome being excellent functional outcome (mRS 0–1) at 90 days. The trial included 516 patients, with 264 randomised to tenecteplase and 252 to standard medical therapy. Primary outcome was achieved in 33% of tenecteplase patients compared with 24.2% in the non-thrombolytic arm (relative risk 1.37 (95% CI 1.04–1.81), *p* = 0.03.). Tenecteplase treatment also resulted in increased rates of reperfusion at 24 h (20.1% vs. 11.8%). The rate of symptomatic intracranial haemorrhage was 3% in the tenecteplase arm, consistent with rates of symptomatic intracranial haemorrhage from other tenecteplase trials.

There is, therefore, a large body of evidence that selection for intravenous thrombolysis can be selected using a tissue clock paradigm rather than a pre-defined rigid time criteria. However, perfusion imaging is not sensitive for lacunar infarcts or brainstem infarcts, and imaging-based approaches for extended-window thrombolysis may only be applicable using MRI.

There is emerging evidence of the use of tenecteplase in the extended time window beyond 4.5 h, but the evidence of clinical benefit is limited to two clinical trials in patients with proximal LVO. The results of TRACE III suggest tenecteplase is reasonable in patients with LVO and perfusion mismatch but without access to thrombectomy, but there is currently no firmevidence to support routine use of tenecteplase beyond the standard 4.5 h time window, either as stand-alone treatment or as bridging treatment prior to thrombectomy.

## 3. Clinical Trials of Tenecteplase

Tenecteplase has been long perceived as a potential thrombolytic agent to replace alteplase. Tenecteplase has several practical and pharmacological advantages to alteplase; it can be administered as a single bolus and has 15-times-higher specificity to fibrin, decreased binding affinity to plasminogen activator inhibitor 1 (PAI−1), and a longer half-life than alteplase. In addition to TIMELESS and TRACE III, there have been a number of randomised clinical trials assessing tenecteplase stroke thrombolysis, and the table below summarises the main studies over the last 8 years. The early clinical trials of tenecteplase used different dosing regimens, from 0.1 mg/kg to 0.5 mg/kg. However, 0.5 mg/kg was associated with significant haemorrhagic risk [15] and this dosing has not been further tested in clinical trials. A summary of clinical trial data is provided in Table 1. The following passages will focus on the more recent clinical trials.

The first positive trial was the Australian Tenecteplase Trial, which randomised patients to one standard-dose alteplase (n = 25) and two tenecteplase dosing arms at 0.1 mg/kg and 0.25 mg/kg (n = 25 in each group) in patients with target CT perfusion mismatch within 6 h of symptom onset [23]. The pooled tenecteplase arms had high rates of reperfusion and clinical improvement at 24 h when compared to alteplase, and the 0.25 mg/kg tenecteplase dose was superior to the lower dose and to alteplase on all efficacy outcomes. This trial marked a significant milestone as it suggested the efficacy of 0.25 mg/kg tenecteplase in recanalizing vascular occlusions.

The Norwegian Tenecteplase Stroke Trial (NOR-TEST) trial was the first phase III tenecteplase stroke thrombolysis trial to be completed. It assessed the efficacy and safety of 0.4 mg/kg tenecteplase compared to the standard dosing of alteplase (0.9 mg/kg) in patients presenting with acute ischaemic stroke [16]. The trial randomised 1100 patients, with 549 to tenecteplase and 555 to alteplase. NOR-TEST, however, failed to show the superiority of 0.4 mg/kg tenecteplase when compared to alteplase for achieving 90-day independence, (64% vs. 63%, odds ratio 1.08, 95% CI 0.84–1·38; *p* = 0.52,), and there was no increase in symptomatic haemorrhage (3% vs. 2%, *p* = 0.49). However, this trial included patients with minor symptoms (median NIHSS 4), and nearly a fifth of the enrolled patients had a stroke mimic diagnosis. A subsequent trial, the NOR-TEST 2A trial, compared 0.4 mg/kg tenecteplase to alteplase and randomised 216 patients before the trial was stopped early due to signs of harm. Treatment with 0.4 mg/kg tenecteplase resulted in increased intracranial haemorrhage (21/100, 21%) when compared to alteplase (7/104, 6.7%), unadjusted OR 3·68 [95% CI 1.49–9.11]; *p* = 0.0031) and higher mortality (15/96, 15.6% vs. 5/101, 5.0%, unadjusted OR 3.56 [95% CI 1.24–10.21]; *p* = 0.013) [5].

The efficacy of tenecteplase in achieving recanalization in the LVO population when given within 4.5 h was examined in the two The Tenecteplase versus Alteplase before Endovascular Therapy for Ischemic Stroke (EXTEND-IA TNK) trials [4,17]. In EXTEND-IA TNK part one (n = 202, 101 patients in each thrombolytic arm), tenecteplase 0.25 mg/kg treatment led to 22% early reperfusion of >50% of affected territory or absence of retrievable thrombus when compared to 10% achieved in alteplase (OR 2.6, 95%CI 1.1–5.9, *p* = 0.02). In EXTEND-IA TNK part two, which randomised 300 patients with LVO referred for EVT to either 0.25 mg/kg or 0.4 mg/kg tenecteplase, reported similar rates of early reperfusion in each dosing arm (19% each) aRR, 1.03 (0.66 to 1.61) *p* = 0.89). There were no differences seen in functional outcomes at 90 days (median mRS 2 vs. 2, aOR 0.96 (0.74 to 1.24) *p* = 0.73) and the safety profiles were similar, but the 0.4 mg/kg dose had statistically non-significantly higher rates of symptomatic intracranial haemorrhage (4.7% vs. 1.3%) than the 0.25 mg/kg arm.

The Alteplase Compared to Tenecteplase in Patients With Acute Ischemic Stroke (AcT) trial in Canada was the first large phase III clinical trial to show non-inferiority of 0.25 mg/kg tenecteplase when compared to alteplase within the 4.5 h time window [6]. AcT included 1600 patients including 816 randomised to tenecteplase and 784 to alteplase. The primary outcome was 90–120 excellent outcome defined as modified Rankin 0–1. Tenecteplase treatment resulted in similar rates of excellent functional outcomes when compared to alteplase (36·9% vs. 34·8%, risk different 2.1%, 95% CI −2.6–6.9), which met the prespecified non-inferiority threshold. Tenecteplase also had comparable rates of symptomatic intracranial haemorrhage (3.4% vs. 3.2%) and 90-day mortality (15.3% vs. 15.4%) when compared to alteplase.

The Tenecteplase Reperfusion Therapy in Acute Ischemic Cerebrovascular Events-Ⅱ (TRACE−2) trial randomised 1430 thrombectomy-ineligible patients to either 0.25 mg/kg tenecteplase (n = 716) or 0.9 mg/kg alteplase (n = 714) with the primary endpoint of 90-day excellent outcome (modified Rankin Scale 0–1). Tenecteplase was non-inferior to alteplase, with primary outcome occurring in 62% tenecteplase vs. 58% alteplase patients (risk ratio 1.07, 95% CI 0.98–1.16). Comparable rates of symptomatic intracranial haemorrhage at 36 h (2% in each arm) and 90-day mortality (7% vs. 5%) were noted [7]. It should be noted that in TRACE−2, a Chinese version (CSPC Recomgen Pharmaceutical (Guangzhou, China) Co., Ltd. (formally Guangzhou Recomgen Biotech Co., Ltd.) of tenecteplase (rhTNK-tPA) was used, although it was noted to have similar pharmacological properties to industry-standard tenecteplase (Boehringer Ingelheim, Germany/Genentech, USA).

The tenecteplase versus alteplase for thrombolysis in patients selected by use of perfusion imaging within 4.5 h of onset of ischaemic stroke (TASTE) trial was the only phase III clinical trial to have utilised perfusion imaging selection and randomised 680 patients to either 0.25 mg/kg tenecteplase (n = 339) or standard-dose alteplase (n = 341). It was a non-inferiority trial and was stopped early due to the release of results of other large clinical trials demonstrating non-inferiority. The primary endpoint of 90-day functional independence was achieved in 57% of tenecteplase patients compared with 55.3% in alteplase. The non-inferiority margin of 0.03 was narrowly missed in the intention to treat the population (standardised risk difference, SRD = 0·03[95%CI: −0.033, 0.10], one-tailed pnon-inferiority = 0·031) but the non-inferiority margin was crossed in the per-protocol population. The symptomatic intracranial haemorrhage rate of 3% in the tenecteplase patients were consistent with that reported in other tenecteplase trials utilising 0.25 mg/kg [21].

Importantly, an updated study level meta-analysis published in the TASTE trial manuscript showed that tenecteplase was superior to alteplase, with a number needed to treat of 25 to achieve an additional functional independence outcome. The results of TASTE, the recent tenecteplase trials, and the updated meta-analysis have provided substantial evidence that 0.25 mg tenecteplase is the current optimal treatment for stroke thrombolysis in the standard time window.

## 4. Mild Ischaemic Stroke

Mild AIS treatment, typically defined as an NIHSS of 5 or less, remains an area of controversy as the clinician needs to balance between the risk of haemorrhagic complications and potential benefits of treatment, given that patient recovery is expected as part of natural history. Three randomised clinical trial have been completed.

Two clinical trials have failed to demonstrate the benefits of alteplase in minor ischaemic strokes. In a randomised, double-blind, double-placebo controlled trial, the PRISMS trial, intravenous alteplase was compared to oral aspirin in patients with minor, non-disabling strokes (NIHSS < 5) presenting within 3 h of symptom onset. The trial was stopped early owing to slow recruitment and included 313 patients in its final analysis, with 156 patients in the alteplase group and 157 in the aspirin group. The primary outcome of favourable outcomes (defined as mRS < 2) at 90 days did not differ between the two study groups (alteplase 78.2%, 122/156; aspirin 81.5%, 128/157) [24]. Symptomatic intracranial haemorrhage occurred in 3.2% of alteplase patients while aspirin-treated patients had symptomatic intracranial haemorrhage.

Following the PRISMS trial, Chen et al. subsequently evaluated alteplase vs. dual anti-platelet therapy (DAPT) in the ARAMIS trial. Patients from 38 Chinese hospitals with acute, minor, non-disabling strokes (NIHSS ≤ 5) presenting with 4.5 h of symptom-onset were randomised to DAPT (aspirin and clopidogrel) vs. intravenous alteplase. The trial included 719 patients with 369 patients assigned to DAPT and 350 to alteplase. The primary outcome of favourable neurological outcome (mRS < 2) at 90 days was achieved in 93.8% of the DAPT group and 91.4% of the alteplase group (indicating the non-inferiority of DAPT with a risk difference of 2.3%, 95% CI −1.5% to 6.2%) [25]. Symptomatic intracranial haemorrhage rate was low in both groups (0.3% DAPT group and 0.9% alteplase group). The results of PRISMS and ARAMIS indicate that intravenous alteplase was not more effective in achieving excellent functional outcome with compared with antiplatelet treatment.

A multicentre, prospective, randomised, open-label, blinded-endpoint, controlled trial of thrombolysis with tenecteplase versus standard of care in the prevention of disability at 3 months in minor ischaemic stroke with proven acute symptomatic occlusion (TEMPO−2) trial has recently been published [20]. This trial randomised patients with mild ischaemic stroke (NIHSS ≤ 5) with either intracranial arterial occlusion or focal perfusion abnormality to receive tenecteplase 0.25 mg/kg or best medical therapy within 12 h of stroke onset. The trial was stopped early for futility after 886 patients were included (tenecteplase 369, non-thrombolytic treatment 454). The primary outcome was a return to baseline functioning on a premorbid modified Rankin scale, and there was no difference between the tenecteplase and non-thrombolytic arms (71.5% vs. 74.8%, RR 0.96, 95% CI 0.88–1.04, *p* = 0.2882).

In the subset of patients with proven vascular or large vessel occlusion, tenecteplase treatment resulted in significantly higher rates of recanalization on repeat imaging between 4 to 8 h after randomisation (any vessel occlusion (TNK = 256, control = 259) 48% vs. 22%, *p* < 0.0001; large vessel occlusion (TNK = 46, control = 40), 48% vs. 13%, *p* = 0.0005) [20]. However, subgroup analysis in these populations did not demonstrate improvements in outcomes with tenecteplase, which is an unexpected finding. The reason for this is unclear, and further post hoc analyses will likely provide insights into these findings.

In a recent large systematic review and meta-analysis comparing the effect of thrombolysis with alteplase and best medical therapy in mild ischaemic stroke (NIHSS ≤5), just over 13,000 patients were included [26]. The study involved patients from 20 studies, including three randomised clinical trials, with 4972 patients receiving thrombolysis compared with 8425 patients receiving best medical therapy. There were no differences between thrombolysis and best medical treatment in terms of improved functional outcome, reduced mortality, or rates of recurrent stroke. However, thrombolysis was associated with a nearly two-fold increase in early neurological deterioration (OR, 1.81 [95% CI, 1.17–2.80]) and symptomatic intracerebral haemorrhage (OR, 7.48 [95% CI, 3.55–15.76]).

Along the same lines, a large observational registry by Seners et al. assessed the impact of added EVT on outcomes after thrombolysis with alteplase in patients with mild ischaemic stroke (NIHSS ≤5) with proven proximal occlusion of the M1 or M2 segments [27]. Added EVT after alteplase was associated with increased odds of an excellent outcome in confirmed proximal (OR = 3.26; 95% CI = 1.67–6.35; *p* = 0.0006) or distal (OR = 1.69; 95% CI = 1.01–2.82; *p* = 0.04) M1 occlusion but was associated with lower odds of a good outcome in patients with M2 occlusion (OR = 0.53; 95% CI = 0.38–0.75; *p* = 0.0003) and increased rates of symptomatic haemorrhage (OR = 4.40; 95% CI = 2.20–8.83; *p* < 0.0001).

These studies, along with the recently published TEMPO−2 trial, suggest that intravenous thrombolysis should not be routinely administered to patients with truly non-disabling mild ischaemic stroke. Specific patient characteristics may potentially influence this decision, such as the presence and location of LVO and whether EVT is being considered. However, the authors recommend that the decision-making process for providing thrombolysis to patients with a low NIHSS should be individualised, and that clinicians should consider refraining from treating patients with truly non-disabling stroke, such as minor sensory symptoms or mild non-disabling facial weakness.

## 5. Non-Advanced Imaging for Wake-Up Stroke

Whether non-advanced imaging can be used to select patients for thrombolysis has been tested in the Safety and efficacy of tenecteplase in patients with wake-up stroke assessed by non-contrast CT (TWIST) trial [19]. TWIST randomised 578 patients within 4.5 h of waking or found with stroke symptoms using non-contrast CT scan to either 0.25 mg/kg tenecteplase (n = 288) or best medical therapy (n = 246), with inclusion of patients with vessel occlusion proceeding to thrombectomy in either study group. The primary end point was ordinal shift on the 90-day modified Rankin scale, which was negative (adjusted OR 1.18 955 CI 0.88–1.58, *p* = 0.27). There were no safety concerns with no difference in mortality (10% vs. 9%) or symptomatic intracranial haemorrhage (2% vs. 1%). Although the primary results were negative, the treatment effect was likely reduced by more control patients (14%) proceeding to thrombectomy than tenecteplase (6%) patients. It also provided important safety data with comparable rates of symptomatic haemorrhage compared to clinical trials utilising advanced imaging for patient selection.

## 6. Thrombolysis Prior to Endovascular Thrombectomy

Demonstrating a benefit for pre-thrombectomy thrombolysis has been debated [28]. Whether intravenous thrombolysis (with tPA or TNK) offered any additional benefit over and above that of endovascular thrombectomy was examined in several clinical trials, without a definitive answer. Dissolution of a thrombo-embolus (by thrombolysis) may make for an easier subsequent clearing of the LVO by thrombectomy. However, it is not without cost (time, financial, and potential adverse effects).

A meta-analysis of six randomised trials has recently evaluated this question. The analysis only included patients presenting with an anterior circulation LVO to a thrombectomy-capable centre. Additional benefit from intravenous thrombolysis (defined as a favourable shift in mRS at 90 days) was only seen in patients who received it within 2 h 20 min from symptom onset. After that, thrombolysis did not afford added benefit over and above that of endovascular thrombectomy [29].

## 7. Guideline Recommendations for Tenecteplase

The European Stroke Organisation and the Heart and Stroke Foundation of Canada guidelines have updated their recommendations for tenecteplase. Both best practice guidelines have issued strong recommendations for 0.25 mg/kg tenecteplase as an alternative to alteplase for thrombolysis-eligible patients within 4.5 h from stroke onset [10,30], while the guidelines from the American Heart Association/American Stroke Association have yet to update the previous guidelines issued in 2019 [31].

## 8. Transitioning to Tenecteplase—Real World Application

The results of clinical trials have shown that 0.25 mg/kg tenecteplase is the standard dose for stroke thrombolysis, and given its pragmatic advantages, it is likely that tenecteplase will be used instead of alteplase for stroke thrombolysis. However, tenecteplase has already been used as a routine thrombolytic medication in several centres in New Zealand, the United States, and Europe prior to the completion of large clinical trials.

In New Zealand, tenecteplase transition occurred early in two comprehensive stroke centres in Christchurch and Wellington and associated rural stroke networks, driven by the lack of consistent access to EVT and demonstration in EXTEND-IA TNK trials of the superiority of tenecteplase in LVO [4,17]. The experience of routine use of tenecteplase has been reported in these networks with contemporaneous alteplase comparators. Both studies reported the same rates of symptomatic intracranial haemorrhage (sICH) of 1.8% in tenecteplase patients, which were lower than in alteplase patients (2.4% in Christchurch and 3.4% in Wellington) [32]. Additionally, in the Wellington study, the door-to-needle time was 10 min shorter in tenecteplase patients.

In France, the TETRIS study group (2021) published a retrospective cohort of 588 large vessel occlusion (LVO) stroke patients treated with tenecteplase prior to EVT [33]. They found a comparable sICH rate of 2.5%. Functional independence at 90 days (mRS ≤ 2) was 47.2%, comparable to outcomes after thrombectomy with alteplase. Tenecteplase also resulted in vessel recanalization in 20.5%, consistent with reported rates from the EXTEND-IA TNK trial.

In the United States, the Ascension Network in Texas were the first network to complete the change, and their results were in line with experience from New Zealand, with comparable safety data and a reduction in door-to-needle time [34]

The largest real-world experience has been reported by The Comparative Effectiveness of Routine Tenecteplase vs. Alteplase in Acute Ischemic Stroke (CERTAIN) collaboration, an academic collaboration from 25 stroke networks with >100 thrombolysis-capable hospitals throughout New Zealand, Australia, and the United States. An analysis of symptomatic intracranial haemorrhage risk between thrombolytic agents included 9238 patients (1925 tenecteplase and 7313 alteplase patients). The tenecteplase-treated group had significantly lower symptomatic haemorrhage risk (1.8% with tenecteplase- vs. 3.6% with alteplase-treated patients, *p* value < 0.001), despite tenecteplase patients having higher risk features for haemorrhage including older age (73 vs. 70), more male sex (54% vs. 51%), higher NIHSS (9 vs. 7), and higher rates of LVO (48% vs. 25%). The lower risk of symptomatic haemorrhage was consistent throughout subgroups with or without EVT [35].

Tenecteplase may also offer a financial benefit when compared to alteplase. A retrospective medical record review across six hospitals over a 4-month period in 2022 in the United States showed a direct cost saving for the health system of $209,476.80 when treating 129 acute ischaemic stroke patients with tenecteplase compared to alteplase (102 patients with alteplase and 117 patients with tenecteplase) [36].

## 9. Pragmatic Consideration for Transitioning to Tenecteplase Thrombolysis

It is anticipated that tenecteplase will progressively transition into routine stroke thrombolysis on a global scale. However, the current tenecteplase vials are available in 40 mg or 50 mg doses with markers designed for cardiac thrombolysis (0.5 mg/kg). Early adopters from New Zealand and the United States have outlined the transition processes, emphasising the need for various clinical stakeholders, including emergency department staff, nursing staff, and general medical personnel managing acute stroke, to be informed of protocol changes [32,34,37]. To prevent potential dosing errors, the authors recommend the use of tenecteplase dosing charts similar to the existing weight-based dosing for alteplase and to clearly indicate that the current dosing markers on the 40 or 50 mg vials are not intended for stroke use to prevent administration of the cardiac dose of tenecteplase. Upon transitioning, it is crucial to remove all alteplase vials from existing drug stocks to mitigate the risk of administering the incorrect thrombolytic agent. It is expected that the production of stroke-appropriate dosing vials will commence alongside the rollout of tenecteplase for stroke care, which will further minimise the potential for dosing errors. Until then, the above strategies will help prevent avoidable errors.

## 10. Tenecteplase Transition to Low Resource Health Settings

A significant proportion of tenecteplase trials have enrolled patients within the standard thrombolysis time window (<4.5 h from last known well or onset time), while other trials have included extended time window studies requiring advanced imaging, such as perfusion imaging. To date, no tenecteplase trials have used MRI perfusion mismatch to guide patient selection. Access to advanced imaging may not be available across all healthcare settings due to resource limitations, but this does not affect patient selection for tenecteplase thrombolysis within the standard time window. The only tenecteplase trial to have used non-advanced imaging was TWIST, which randomised patients with mild to moderate ischaemic stroke to tenecteplase within 4.5 h of waking up with stroke symptoms based on non-contrast CT selection. Although the trial yielded negative results, the rate of symptomatic intracerebral haemorrhage in the tenecteplase arm was 2%, consistent with rates reported in other tenecteplase clinical trials.

The ongoing EXIST-BT2 clinical trial is randomising 1250 Chinese patients to either tenecteplase or medical management in the 4.5 to 6 h time window using non-advanced imaging selection [38]. Until further evidence is available, the authors believe it is reasonable to consider individualised decision-making to offer tenecteplase thrombolysis for patients with mild to moderate stroke within 4.5 h of waking, provided they have a favourable non-contrast CT scan.

## 11. Conclusions and Future Directions

The landscape of intravenous thrombolysis in acute ischaemic stroke has undergone significant revolutionary changes over recent years. The window of eligibility has continued to expand with the use of advanced imaging, transitioning into an era utilising the tissue clock instead of a rigid time clock for patient selection. More recent trials have proven that 0.25 mg/kg tenecteplase is non-inferior to alteplase and is superior to alteplase at achieving vascular recanalization. The recent trial data will result in a global changing of the guard to tenecteplase.

Despite the advantages, there remains a number of unaddressed questions in tenecteplase thrombolysis including extrapolating results from alteplase such as utilising MRI DWI-FLAIR mismatch for selection of patients with unknown onset time. In low-income regions of the world, where access to advanced imaging is limited, could basic imaging modalities such as non-contrast CT be sufficient for patient selection? Finally, given the improving work flow in reducing time metrics for patients proceeding to thrombectomy, does bridging therapy with tenecteplase improve patient outcome? These questions are likely to be addressed with further real world data and results of ongoing clinical trials to optimise patient management and outcome with thrombolysis for acute ischaemic stroke.

## Figures and Tables

**Table 1:** Recent studies investigating the use of tenecteplase in acute stroke management.

Author	Year	Design/Trial Name	Participants	Tenecteplase dose	Comparison	Primary Outcome	Notes
Logallo et al. [16]	2017	Randomised, open-label, blinded, superiority trial. NOR-TEST.	1100, across 13 stroke units in Norway	0.4 mg/kg	Alteplase 0.9 mg/kg	mRS 0–3 at 3 months. OR 1.08 (CI 0.84–1.38) *p* = 0.52	Within 4.5 h of symptom onset of awakening with symptoms. Included bridging to thrombectomy. Median NIHSS 4 (IQR 2–8). Secondary and safety outcomes: -Death at day 90 (*p* = 0.68) -Serious adverse effects by day 90 (*p* = 0.74)
Campbell et al. [4]	2018	Randomised, open-label, blinded, non-inferiority followed by superiority trial. EXTEND-IA TNK part 1.	202, across 13 centres in Australia and New Zealand	0.25 mg/kg	Alteplase 0.9 mg/kg	Reperfusion of >50% ischaemic territory, or absence of retrievable clot. Non inferiority: -Incidence difference 12 percentage point (CI 2–21) -*p* = 0.002) Superiority: -Adjusted OR 2.6 (1.1–5.9) -*p* = 0.02.	Within 4.5 h of symptom onset. Included bridging to thrombectomy. Large vessel occlusions (ICA, M1, M2, basilar). Median NIHSS 17 (IQR 12–22) both groups. Secondary and safety outcomes: -mRS ordinal at 90 days (TNK 2 vs. 3 *p* = 0.04) -Functional independence (*p* = 0.06) -Early neuro improvement (*p* = 0.70) -Safety death (*p* = 0.08) -sICH (*p* = 0.99)
Campbell et al. [17]	2020	Randomised, open-label, blinded. EXTEND-IA TNK part 2.	300, across 27 hospitals in Australia and New Zealand	0.4 mg/kg	0.25 mg/kg tenecteplase	Reperfusion of >50% ischaemic territory. Risk difference 0.0% (CI −8.9%–8.9%) *p* = 0.89.	Within 4.5 h of symptom onset, before planned thrombectomy. Large vessel occlusions (ICA, M1, M2, basilar). Median NIHSS 17 (0.4 mg/kg) and 16 (0.25 mg/kg). Secondary and safety outcomes: -mRS 90 days (*p* = 0.73) -Freedom from disability (*p* = 0.69) -sICH 36 h (*p* = 0.12) -All-cause death (*p* = 0.35)
Bivard et al. [18]	2022	Randomised, open-label, blinded (masked), superiority. TASTE-A.	104 across 5 tertiary Melbourne hospitals	0.25 mg/kg	Alteplase 0.9 mg/kg	Volume of perfusion lesion on arrival to hospital on CTP. Adjusted incidence rate ratio 0.55 (CI 0.37–0.81) *p* = 0.003	Within 4.5 h of symptom onset. Median NIHSS 8 both groups (IQR 5–14 and 5–17). Secondary and safety outcomes: -mRS 5–6 at 90 days (*p* = 0.93) -sICH 36 h (none occurred) -Death 90 days (*p* = 0.88)
Kvistad et al. [5]	2022	Randomised, open-label, blinded, non-inferiority (3% margin). NOR-TEST 2, part A.	204 patients across 11 hospitals in Norway	0.4 mg/kg	Alteplase 0.9 mg/kg	mRS 0–1 at 3 months. OR 0.45 (CI 0.25–0.8). *p* = 0.0064	Within 4.5 h of symptom onset. Stopped early due to higher sICH rates in TNK group. Moderate or severe strokes, NIHSS 6 or more. Secondary and safety outcomes: -Any ICH (more TNK *p* = 0.0031) -sICH (more TNK *p* = 0.061) -Mortality (more TNK *p* = 0.013)
Menon et al. [6]	2022	Randomised, open-label, blinded, non-inferiority 5% margin (secondary superiority). AcT.	1577 patients across 22 primary and comprehensive stroke centres in Canada	0.25 mg/kg	Alteplase 0.9 mg/kg	mRS 0–1 at 90–120 days. Risk difference 2.15 (CI −2.6–6.9). Meeting non-inferiority threshold.	Within 4.5 h of symptom onset. Included bridging to thrombectomy. TNK trend to better, not superior, on secondary analysis. Median NIHSS 9 (TNK) 10 (alteplase). Secondary and safety outcomes): -sICH (no difference) -death (no difference)
Roaldsen et al. [19]	2023	Randomised, control, open-label, blinded endpoint. TWIST.	578 patients across 77 hospitals in 10 countries.	0.25 mg/kg	No thrombolysis	mRS at 90 days (ordinal logistic regression with ITT). OR 1.18 (CI 0.88–1.58). *p* = 0.27.	Within 4.5 h of awakening with symptoms. Wake-up stroke NIHSS > 2 or aphasia. Selection with non-contrast CT. Median ASPECT 10. >50% NIHSS < 8 (see table). Secondary and safety outcomes: -Mortality (*p* = 0.37) -sICH (*p* = 0.28) -Any intracranial haem (*p* = 0.64)
Wang et al. [7]	2023	Randomised, open-label, blinded endpoint, non-inferiority 3.74%. TRACE−2.	1430 patients across 53 centres in China	0.25 mg/kg	Alteplase 0.9 mg/kg	mRS 0–1 at 90 days. RR 1.07 (CI 0.98–1.16). TNK non-inferior.	Within 4.5 h of last known well. Excluded if thrombectomy candidate (ineligible or refused). NIHSS 5–25. >50% NIHSS < 8. Secondary and safety outcomes: -sICH (*p* = 0.74) -Mortality (0.22)
Albers et al. [14]	2024	Randomised placebo control, double-blind. TIMELESS.	458 patients across from 112 centres across USA and Canada.	0.25 mg/kg	Placebo	mRS at 90 days. Adjusted common odds ratio 1.13 (CI 0.82–1.57). *p* = 0.45.	4.5–24 h since last known well. Bridging to thrombectomy included (77.3% of patients). MCA M1 or M2 or ICA ~only. Median NIHSS 12 both groups. Secondary and safety outcomes: -Functional independence (no difference) -sICH (no difference) -Death (no difference) -Sub-group analysis (not powered) favoured TNK in M1 occlusion.
Coutts et al. [20]	2024	Randomised, open-label control trial, TEMPO−2	886 patients across 48 hospitals in Australia, Austria, Brazil, Canada, Finland, Ireland, New Zealand, Singapore, Spain, and UK.	0.25 mg/kg	Non-thrombolytic standard of care.	Return to baseline function (mRS) RR 0.96 (CI 0.88–1.04) *p* = 0.29	Within 12 h of stroke onset. Stopped early for futility (no benefit and possible harm). Minor stroke NIHSS 0–5 with vessel occlusion or perfusion deficit on imaging. Secondary and safety outcomes: -sICH—higher in TNK: RR 4.2 (0.9–19.7, *p* = 0.059) -Death—higher in TNK: adjusted HR 3.8 (CI 1.4–10.2, *p* = 0.0085)
Xiong et al. [9]	2024	Randomised blinded end-point evaluation control, open-label trial TRACE-III	516 patients across 58 centres in China.	0.25 mg/kg	Standard medical treatment.	Absence of disability (mRS 0–1) at 90 days Relative rate (?OR) 1.37 (CI 1.04–1.81) *p* = 0.03	4.5–24 h from last known well. Large vessel occlusion (ICA or MCA branches M1 or M2). Excluded if planned for thrombectomy, but <2% (similar in each group) had rescue thrombectomy. Median NIHSS 11 in TNK and 10 in controls groups. Secondary and safety outcomes: -sICH higher in TNK group. -Death (similar between groups)
Parsons et al. [21]	2024	Randomised, open-label, blinded endpoint, non-inferiority 3%. TASTE	601 patients (of planned 830 patients) across 35 hospitals in 8 countries.	0.25 mg/kg	Alteplase 0.9 mg/kg	mRS 0–1 at 3 months. Standardised RD 0.03 (non-inferiority criteria less than −0.03)	Within 4.5 h of symptom onset. Stopped early due to results of previous tenecteplase trials. Non-inferiority demonstrated on per-protocol analysis. Safety Secondary and safety outcomes: -sICH -All cause mortality
Muir et al. [22]	Yet to be published. Preprint in the Lancet	Randomised, non-inferior and superiority. ATTEST−2.	1858 patients across 40 hospitals in the UK.	0.25 mg/kg	Alteplase 0.9 mg/kg	Adjusted common OR 1.07 (CI 0.90–1.27) meeting non-inferiority, but not superior.	Not published yet but presented at World Stroke Conference 2023. Within 4.5 h of symptom onset. Secondary and safety outcomes: -mRS 0–1 -mRS overall -Safety (no difference)

CI, 95% confidence interval; ICH, intracerebral haemorrhage; IQR, interquartile range; mRS, modified Rankin Scale; NIHSS, National Institutes of Health Stroke Scale; OR, odds ratio; RD, risk difference; RR, relative risk; TNK, Tenecteplase; sICH, symptomatic intracerebral haemorrhage.

## Data Availability

Data sharing is not applicable.
